# The association of hypernatremia and hypertonic saline irrigation in hepatic hydatid cysts: A case report and retrospective study

**DOI:** 10.1097/MD.0000000000007889

**Published:** 2017-09-15

**Authors:** Rujun Zeng, Renhua Wu, Qingguo Lv, Nanwei Tong, Yuwei Zhang

**Affiliations:** a Department of Obstetrics and Gynecology, West China Second University Hospital; b Department of Endocrinology and Metabolism, West China Hospital, Sichuan University, Chengdu, China.

**Keywords:** hepatic hydatid disease, hypernatremia, hypertonic saline

## Abstract

**Rationale:**

Hypernatremia is a rare but fatal complication of hypertonic saline (HS) irrigation in hepatic hydatid disease. It needs careful monitoring and treatment.

**Patient concerns:**

A 28-year-old woman with hepatic hydatid cysts who received operation treatment developed electrolyte disturbances. We also conducted a retrospective study about influence of HS application on electrolytes in patients with hepatic hydatid disease receiving surgery.

**Diagnoses:**

Hypernatremia, developed after HS irrigation.

**Interventions:**

Normal saline, 5% dextrose and other supportive treatment were administered. In the retrospective study, a comparison of electrolyte and glucose fluctuation was made among different HS application groups.

**Outcomes:**

The patient developed hypernatremia after irrigation with HS and died from severe complications. Although some cases of complications are found, no significant relationship between HS irrigation and hypernatremia was reported according to the retrospective study.

**Lessons:**

Hypernatremia after HS irrigation remains rare but might cause severe complications. Monitoring and appropriate treatment are needed to improve prognosis.

## 1 Introduction

Hydatid disease is a zoonotic infectious disease mainly caused by *Echinococcus granulosus* and *Echinocossus multilocularis*. Previous studies reported that the prevalence of hydatid disease is from 0.5% to 6.5%.^[1]^

According to the expert consensus in 2010,^[2]^ the evidence of best treatment option is lack. Generally, antiparasitic treatment, percutaneous treatment, or surgery could be considered. For surgery and percutaneous treatment, protoscolicide is needed to avoid recurrence due to cyst content residues or spillage. Formalin has been rarely used for the risk of death from acidosis and obliterative cholangitis.^[3]^ Silver nitrate could lead to biliary epithelium injury.^[4]^ The HS, from 3% to 30%, is recommended by WHO/OIE and has been used for a long time.^[5]^

Hypernatremia is defined as the plasma sodium level over 145 mM and is associated with severe mortality (40–60%).^[6]^ For patients with acute hypernatremia, they might present with less responsive, dysphagia, shortness of breath, vomiting, and so on.^[7]^ Iatrogenic hypernatremia, although rare, might develop after HS was applied as the scolicidal agent. Here, we reported a case with fatal hypernatremia after hepatic hydatid cyst surgery and made a review of hepatic hydatid cases in our hospital during the last 5 years.

## 2 Case report

A 28-year-old woman was admitted to hospital complaining of left upper quadrant abdominal distension for over 7 months and weight loss of 8 kg. Blood examinations did not find anything abnormal. The computed tomography findings were cystic and low density image of about 9 cm × 8 cm in size, with sharp rims and septations inside. So it was CE2 according to the WHO classification.^[8]^ The cyst mainly occupied the right lobe and was close the tight hepatic vein. A giant mass was observed on the upper part of right hepatic lobe during the laparoscopic surgery. Much yellow viscous fluid and colloid cyst contents were found after opening the pericystic membrane. After aspiration of the cyst contents, 300 mL 20% hypertonic saline (HS) was injected into the cyst cavity and left for 5 minutes. The fluid and cyst contents were carefully removed, followed by irrigation of 20% HS and normal saline into the cyst cavity for 5 minutes, respectively. Histopathological examination of the cyst wall confirmed it was echinococciasis.

Half an hour later, the blood pressure (BP) decreased and blood examination showed abnormalities (sodium: 188.8 mmol/L, potassium: 2.78 mmol/L, glucose: 18.22 mmol/L). Hypotonic fluid and 5% destrose were used to irrigate abdominal cavity. Potassium chloride, dextrose, and normal saline were intravenously infused and the sodium decreased to 183 mmol/L. The patient was transferred to the intensive care unit after central vena catheterization was applied.

**Figure 1:**
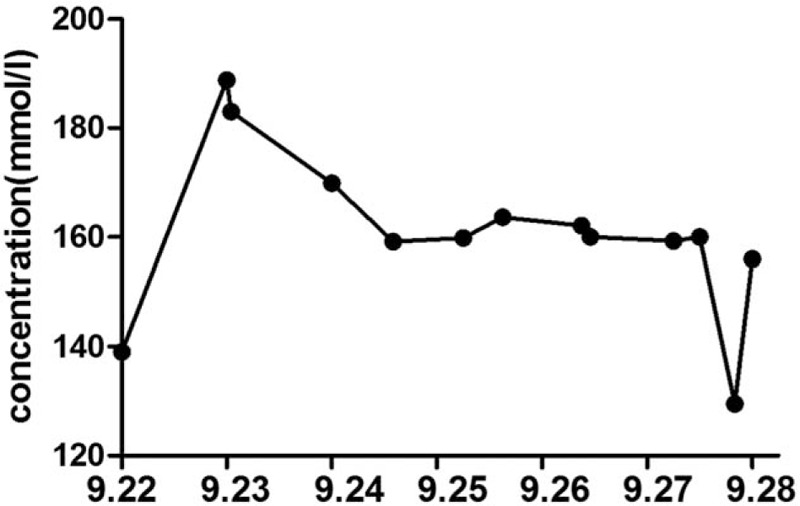
Time course of sodium concentration pre- and postoperation.

**Figure 2:**
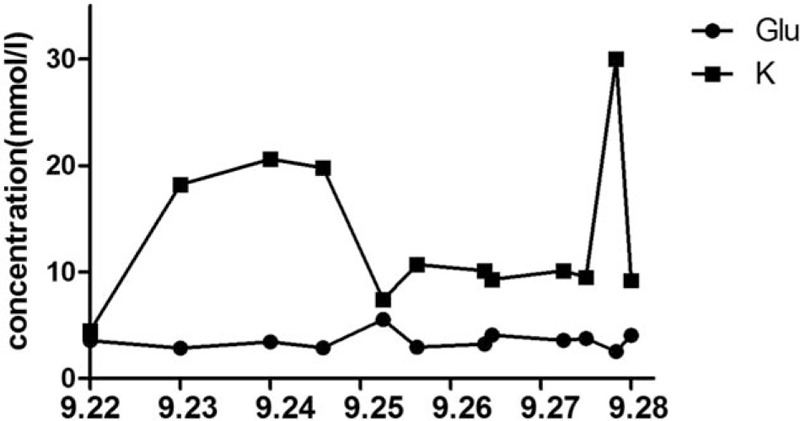
Time course of potassium and glucose concentration pre- and postoperation.

**Figure 3:**
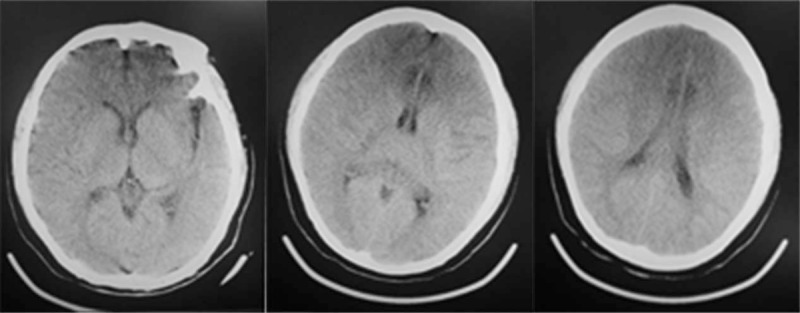
Cranial CT showing diffuse low density image, suggesting cerebral edema. CT = computed tomography.

The patient remained unconscious with no reaction to painful stimuli in the ICU and with the following findings: BP: 115/59 mm Hg; heart rate: 88 bpm; respiratory rate: 24 bpm; body temperature: 38.8°C. We administered hibernation mixture and performed a blood culture (results: negative). Blood laboratory tests revealed acidosis (pH: 7.306, partial pressure of oxygen: 125.2, partial pressure of carbon dioxide: 34.0, bicarbonate: 15.5 mm Hg, lactate: 4.9 mmol/L), coagulopathy (prothrombin time (PT): 16.9 s, activated partial thromboplastin time (APTT): 88.2 s, fibrinogen (FIB): 1.83 g/L, antithrombin III 68.9%, fibrinogen degradation products: > 80 mg/L, D-dimer 38.00 mg/L FEU), and hepatic damage (alanine aminotransferase 76 IU/L, aspartate transaminase: 179 IU/L). The patient also presented with convulsions, and diazepam was administered. Normal saline and 5% dextrose were administered intravenously without affecting hematological abnormalities (sodium: 169.9 mmol/L, potassium: 3.44 mmol/L, calcium: 0.98 mmol/L, and glucose: 20.6 mmol/L) (Figs. 1 and 2). Based on these findings, potassium chloride solution, insulin, and lactated ringers solution were added. The patient's pupils were unequal in size and pupillary reactions to light were absent the day after surgery. Cranial computed tomography (CT) revealed a diffused low-density area (Fig. 3). We then administered a combination of mannitol and furosemide, and because the patient was hemodynamically unstable, epinephrine was also given. Five days postoperatively, sodium decreased to 129.5 mmol/L, calcium decreased to 0.79 mmol/L, and glucose increased to 30 mmol/L, suddenly, and the patient died a few hours later because of central circulatory and respiratory failure and multiple organ failure.

## 3 Method

We retrospectively collected patients diagnosed as hepatic hydatid disease and received surgical treatment from January 1, 2009, to August 31, 2016, in West China Hospital. The diagnosis was certified by the pathological examination. Patients receiving HS irrigation were included and classified according to the concentration and time duration of HS.

The clinical data, including plasma and HS related parameters, was collected from electronic records. If the HS was used more than once, we counted the total duration. The data included fasting blood-glucose (FBG), electrolyte, platelet, coagulation indicators (PT, APTT, and so on) pre- and postoperation, and concentration and time duration of HS during operation. Thus, the fluctuations of these plasma parameters were calculated. Hypernatremia was diagnosed as the plasma sodium level over 145 mmol/L. Neurological imaging was applied to help estimate the intracranial changes.

The analyses were conducted using SPSS 22(SPSS, Inc., Chicago, IL). Continuous variables followed normal distributions were expressed as mean ± standard deviation and were compared by analysis of variance (ANOVA). Categorical data were compared by the chi-square test. Data followed abnormal distributions were expressed as median (interquartile range) and were compared by the Kruskal–Wallis test. *P* value of < .05 was considered to be statistically significant. The ethical statement was not required in this research.

## 4 Result

### 4.1 Study group characteristics

A total of 80 patients were included in this study and were divided into 7 groups. We defined group 1, 2, 3, 4, and 5 as 10%HS for 5 minutes, 10 minutes, 15 minutes, 20 minutes, and 30 minutes, and defined group 6 and 7 as 20%HS for 10 minutes and 25% for 10 minutes, respectively. There were no comparable differences in pre-operation demographics between the groups (Table 1), and the coagulation function was normal in all patients.

**Table 1:**
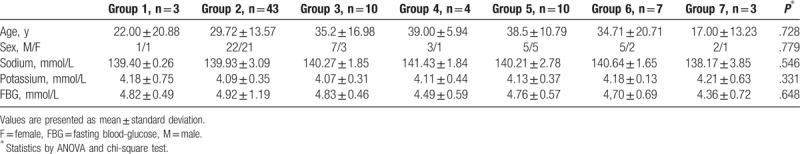
Demographics of patients with hypatic hydatid disease pre-operation.

### 4.2 Relationship between hypertonic saline utility and electrolyte fluctuation

We analyzed the pre- and postoperative electrolyte fluctuations and found no statistically significant difference in changes in the plasma parameters among the 7 groups (Table 2).

**Table 2:**
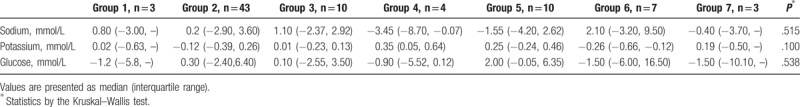
Comparison of electrolytes and glucose change among the 7 groups.

It is important to note that 2 cases, one from group 2 and another from group 6, had dramatically elevated plasma sodium concentrations. The patient from group 2 had a sodium increase of 50.1 mmol/L (postoperative level: 190.0 mmol/L), and she presented unconsciousness with a partial pressure of oxygen of 55 mm Hg and a heart rate of 55 bpm. Cranial CT suggested parenchymal swelling. The patient from group 6 had a sodium increase of 53.4 mmol/L (postoperative level: 193.4 mmol/L) and a glucose increase of 39.16 mmol/L. This patient suffered supraventricular arrhythmia and unstable BP with abnormal coagulation function (fibrinogen degradation products: > 80 mg/L, D-dimer: 17.98 mg/L, FIB: 0.65 g/L, platelets 85 × 109/L, PT: 58.9 s, APTT: 113.9 s). Diffuse parenchymal swelling and subarachnoid hemorrhage were seen on cranial CT. Although a patient from group 3 was not diagnosed as hypernatremia, she presented with coagulopathy (PT 16.3 s, APTT 51 s, FIB 1.01 g/L).

## 5 Discussion

Surgery is the first-line choice of complicated cysts, which contains CE2 (WHO classification) of this case according to the WHO experts and an expert consensus in 2010.^[2,9]^ The percutaneous treatment (PAIR) is not suitable for multiseptated cyst and has a risk of recurrence. So after communicating with the patients, the laparoscopic subtotal pericystectomy had been chosen for the treatment. The lesion was found to be close the right hepatic vein, so the surgeons completely removed the cyst contents and partly removed the pericystic membrane. The hypertonic saline was used as scolicidal agents to prevent recurrence. However, the patient developed hypernatremia after HS irrigation and showed cerebral edema, coagulation dysfunction, electrolyte disturbance, and acid-base imbalance. Hypernatremia was the underlying and highest-priority problem in this patient. The fluid transferred from intracellular to extracellular and resulted in pulmonary edema and brain dehydration. The later leaded to brain vascular rupture and neurological damage. Inappropriate extracellular osmotic tonicity correction could result in central nervous system swelling, which also contributed to this patient's brain injury. The sodium imbalance also contributed to the coagulation dysfunction. Similar abnormalities were reported by Maria in which the patient developed thrombi in the coronary sinus secondary to acute hypernatremia.^[10]^ The mechanism underlying the hypercoagulability remains unclear. Bouchama et al^[11]^ revealed that the von Willebrand factor produced by vascular endothelial cells could be stimulated by elevated plasma sodium concentration. High electrolyte concentration and relative dehydration might also activate intrinsic coagulation, leading to microthrombus formation and activating fibrinolysis.^[10]^

The first case reporting acute hypernatremia following hepatic hydatid surgery was reported in 1982.^[12]^ During the surgery, the tissue around the lesion was carefully protected and there was no fluid spillage. We considered that the HS might have been absorbed through the cyst walls or peritoneal membrane, and in rare cases, the HS was directly inappropriately injected into the hepatic blood vessels. So care is needed when irrigating hydatid cysts with HS, and the duct connecting the cyst and biliary tract should be controlled before HS administration. Close intra- and postoperative electrolyte monitoring is also important.

Acute hypernatremia should be treated carefully, with the principle that the correction rate of the serum sodium level is limited to 8 mmol/L in the first 24 hours and 18 mmol/l in the first 48 hours. In the early period, sodium decreasing at 1 to 2 mmol/L/h is acceptable and improves symptoms.^[13]^ Isotonic intravenous fluids could also restore hemodynamics. Intracellular fluid volume and osmolarity may be corrected by 5% dextrose infusion, and furosemide is recommended to prevent water intoxication resulting from inappropriate correction of electrolyte levels.^[14]^ In our case, we were unable to reduce the sodium concentration properly, which deteriorated the patient's condition.

Animal experiments have shown that increasing the HS concentration was related to electrolyte imbalance and a poor prognosis.^[15]^ Data regarding the influence of HS irrigation on serum electrolytes in humans is lacking. Our retrospective study showed no significant difference among different HS applications, although we reported a small number of high-risk cases. However, our results must be interpreted with caution because of the small sample size. We also speculated that patients might have concurrent abnormal glucose concentration and hypernatremia. Therefore, it is important to balance the administration of insulin and dextrose. Also, patients with normal postoperative sodium concentrations may also have coagulation abnormalities. The reasons might include anaphylaxis after spilling of cystic contents, transfusion reaction, and other unknown factors.

In conclusion, to the best of our knowledge, this is the first case reporting coagulation dysfunction and glucose fluctuation secondary to severe acute hypernatraemia resulting from the hypertonic saline irrigation. As an effective scolicidal agent, HS has a rare risk of developing hypernatremia and should be used with great caution. It is important to keep close monitoring of these relevant disturbances above in the perioperative period. There was no significant difference among every kind of HS application on sodium fluctuation. The most appropriate concentration of HS has not been suggested.
