# The association of hypernatremia and hypertonic saline irrigation in hepatic hydatid cysts: A case report and retrospective study: Erratum

**DOI:** 10.1097/MD.0000000000009528

**Published:** 2017-12-29

In the article, “The association of hypernatremia and hypertonic saline irrigation in hepatic hydatid cysts: A case report and retrospective study”^[1]^, which appeared in Volume 96, Issue 37 of *Medicine*, Rujun Zeng's affiliation should be “Department of Obstetrics and Gynecology, West China School of Medicine, Sichuan University, Chengdu, China.”
