# The association of hypernatremia and hypertonic saline irrigation in hepatic hydatid cysts: A case report and retrospective study: Erratum

**DOI:** 10.1097/MD.0000000000009739

**Published:** 2018-01-26

In the article, “The association of hypernatremia and hypertonic saline irrigation in hepatic hydatid cysts: A case report and retrospective study”,^[1]^ which appeared in Volume 96, Issue 37 of *Medicine*, Rujun Zeng's affiliation should be “West China School of Medicine, Sichuan University, Chengdu, China” and was incorrectly changed in the previous erratum.
